# Personalized risk for clinical progression in cognitively normal subjects—the ABIDE project

**DOI:** 10.1186/s13195-019-0487-y

**Published:** 2019-04-16

**Authors:** Ingrid S. van Maurik, Rosalinde E. R. Slot, Sander C. J. Verfaillie, Marissa D. Zwan, Femke H. Bouwman, Niels D. Prins, Charlotte E. Teunissen, Philip Scheltens, Frederik Barkhof, Mike P. Wattjes, Jose Luis Molinuevo, Lorena Rami, Steffen Wolfsgruber, Oliver Peters, Frank Jessen, Johannes Berkhof, Wiesje M. van der Flier, for the Alzheimer’s Disease Neuroimaging Initiative

**Affiliations:** 1 Alzheimer Center Amsterdam, Department of Neurology, Amsterdam Neuroscience, Vrije Universiteit Amsterdam, Amsterdam UMC, Amsterdam, The Netherlands; 2 Department of Epidemiology and Biostatistics, Vrije Universiteit Amsterdam, Amsterdam UMC, Amsterdam, The Netherlands; 3 Brain Research Center, Amsterdam, The Netherlands; 4 Neurochemistry Laboratory and Biobank, Department of Clinical Chemistry, Amsterdam Neuroscience, Vrije Universiteit Amsterdam, Amsterdam UMC, Amsterdam, The Netherlands; 5 Department of Radiology and Nuclear Medicine, Amsterdam Neuroscience, Vrije Universiteit Amsterdam, Amsterdam UMC, Amsterdam, The Netherlands; 6 Institutes of Neurology and Healthcare Engineering, UCL, London, UK; 7 Alzheimer’s Disease and Other Cognitive Disorders Unit, Neurology Service, Hospital Clínic, Barcelona, Spain and Institut d’Investigacions Biomèdiques August Pi i Sunyer (IDIBAPS), Barcelona, Spain; 8 Department of Psychiatry and Psychotherapy, University of Bonn, Bonn, Germany; 9 German Center for Neurodegenerative Diseases, Bonn, Germany; 10 Department of Psychiatry, Charité Berlin, Campus Benjamin Franklin, Berlin, Germany; 11 Department of Psychiatry, University of Cologne, Cologne, Germany

**Keywords:** Biomarkers, Progression, Cerebrospinal fluid, Magnetic resonance imaging

## Abstract

**Background:**

Biomarkers such as cerebrospinal fluid (CSF) and magnetic resonance imaging (MRI) have predictive value for progression to dementia in patients with mild cognitive impairment (MCI). The pre-dementia stage takes far longer, and the interpretation of biomarker findings is particular relevant for individuals who present at a memory clinic, but are deemed cognitively normal. The objective of the current study is to construct biomarker-based prognostic models for personalized risk of clinical progression in cognitively normal individuals presenting at a memory clinic.

**Methods:**

We included 481 individuals with subjective cognitive decline (SCD) from the Amsterdam Dementia Cohort. Prognostic models were developed by Cox regression with patient characteristics, MRI, and/or CSF biomarkers to predict clinical progression to MCI or dementia. We estimated 5- and 3-year individualized risks based on patient-specific values. External validation was performed on Alzheimer’s Disease Neuroimaging Initiative (ADNI) and an European dataset.

**Results:**

Based on demographics only (Harrell’s *C* = 0.70), 5- and 3-year progression risks varied from 6% [3–11] and 4% [2–8] (age 55, MMSE 30) to 38% [29–49] and 28% [21–37] (age 70, MMSE 27). Normal CSF biomarkers strongly decreased progression probabilities (Harrell’s *C* = 0.82). By contrast, abnormal CSF markedly increased risk (5 years, 96% [56–100]; 3 years, 89% [44–99]). The CSF model could reclassify 58% of the individuals with an “intermediate” risk (35–65%) based on the demographic model. MRI measures were not retained in the models.

**Conclusion:**

The current study takes the first steps in a personalized approach for cognitively normal individuals by providing biomarker-based prognostic models.

**Electronic supplementary material:**

The online version of this article (10.1186/s13195-019-0487-y) contains supplementary material, which is available to authorized users.

## Background

Dementia disorders place a huge burden on society and are set to bulge due to an aging population [1, 2]. Alzheimer’s disease (AD) is the most common cause of dementia and represents the largest unmet medical need in neurology [3]. In order to bring therapy and support to individuals as timely and accurate as possible, diagnostic tests play a key role. In individual patients with mild cognitive impairment (MCI), biomarkers such as cerebrospinal fluid (CSF) and magnetic resonance imaging (MRI) have been shown to have predictive value for progression to dementia [4]. However, the pre-dementia stage of AD takes far longer, as neuropathological changes already start in the cognitively normal stage [3, 5–8]. For that reason, recent criteria proposed a biological framework for AD in which AD is classified based on the presence of pathology rather than the presence of clinical symptoms [8].

On a group level, AD biomarkers are predictive in cognitively normal individuals as well. For example, a reduced hippocampal volume on MRI has been associated with an increased risk of clinical progression [9–11]. Furthermore, an abnormal AD biomarker profile in CSF has been shown to be strongly associated with clinical progression [12–16]. Moreover, even relatively low amyloid β1–42 (Aβ) levels, yet within the normal range, have been associated with clinical progression, indicating that simple dichotomous cutoffs fail to extract all information available in these markers and moreover may erroneously reassure individuals [17].

The question how these findings on group level translate to the individual is particular relevant for individuals who present with worries about their memory at a memory clinic, but are deemed cognitively normal. Unfortunately, findings on group level cannot be translated directly to the individual and the interpretation of biomarkers is not optimized. In addition, the meaning of biomarkers should ideally be interpreted in the context of an individual’s own characteristics [18], but information on how to weigh and combine multiple sources of information is lacking. Therefore, clinicians are generally reluctant to disclose biomarker results to cognitively normal individuals. Nonetheless, individuals and caregivers become increasingly assertive, demanding more specific and individually tailored information [19].

The objective of this study was to optimize the interpretation of biomarkers by composing individualized prediction models for clinical progression to MCI or dementia based on MRI and/or CSF biomarkers that could be used in cognitively normal individuals.

## Methods

### Participants

We included 481 cognitively normal individuals from the Amsterdam Dementia Cohort (ADC) and ongoing SCIENCe project, with a baseline diagnosis of subjective cognitive decline (SCD), available baseline MMSE, and available baseline MRI and/or CSF data [20, 21]. All individuals had their baseline visit in our memory clinic between January 2000 and November 2015. Individuals with a diagnosis of MCI or dementia within 6 months after baseline were excluded from the analysis as they were likely to have been misclassified at baseline.

Baseline diagnostic work-up consisted of a standardized 1-day dementia screening [20, 21]. Clinical diagnosis was made by consensus in a multi-disciplinary meeting. Individuals were labeled with SCD if they presented with cognitive complaints, had normal results on clinical assessments, and did not meet criteria for MCI, dementia, or any other neurologic or psychiatric disorder known to cause cognitive complaints (i.e., cognitively normal) [22].

Standardized annual follow-up included a follow-up visit with the neurologist and neuropsychologist, and diagnoses were re-evaluated in a multi-disciplinary meeting [20]. Until early 2012, MCI was diagnosed according to Petersen’s criteria and from 2012 onwards, the diagnosis of MCI was based on National Institute on Aging-Alzheimer’s Association (NIA-AA) criteria [23, 24]. The diagnosis of AD dementia and other types of dementia was based on international diagnostic or research consensus criteria [25–28].

### MRI

Before 2008, brain MRI was performed on 1.0 and 1.5T MRI systems (Siemens Magnetom Avanto, Vision, Impact and Sonata, GE Healthcare Signa HDXT). From 2008 on, MRI of the brain was performed on 3T MRI systems (MR750, GE Medical Systems, Ingenuity TF PET/MR, Philips Medical Systems; Titan, Toshiba Medical Systems). The standard dementia protocol with whole brain coverage included near-isotropic sagittal 3D T1-weighted images (including oblique coronal reconstructions), sagittal 3D T2-weighted fluid-attenuated inversion recovery (FLAIR) (including axial reconstructions), axial T2-weighted turbo spin-echo, and axial T2*-weighted gradient echo sequence or alternatively SWI sequences. MRI data was available for 432 (90%) individuals.

Bilateral hippocampal volume (HCV, mL) was estimated using FMRIBs Integrated Registration and Segmentation Tool (FIRST) [29]. Normalized brain volumes (NWBV, mL) were estimated with SIENAX (Structural Image Evaluation using Normalization of Atrophy Cross-sectional) using optimized settings [30]. Additionally, visual rating of MRI was performed according to semi-quantitative visual rating scales for medial temporal lobe atrophy (MTA, 0–4) and global cortical atrophy (GCA, 0–3) [31, 32].

### CSF analysis

CSF was obtained by lumbar puncture and collected in polypropylene tubes (Sarstedt, Nurmberg, Germany) and processed according to international guidelines [33]. CSF biomarkers Amyloid β1–42 (Aβ) and total Tau (tau) were measured using sandwich enzyme-linked immuno sorbent assay (ELISA) on a routine basis (Innotest, Fujirebio, Gent, Belgium) [34]. Baseline CSF data was available for 344 (72%) individuals.

### Statistical analysis

All analyses were carried out in STATA 14SE. Prognostic models were constructed with Cox regression analysis (determinants as continuous measures; CSF biomarkers log-transformed). The models were constructed with complete cases only, and therefore, the number of individuals varied across models. No differences in demographic characteristic or baseline survival were found between individuals with complete data and incomplete data (Additional file 1: Table S1). The clinical end-point was MCI or dementia [23–28].

First, a prognostic model based on patient characteristics (age, gender, and MMSE) and interactions between the characteristics was constructed. Subsequently, we added either MRI biomarkers (volumetric measures: HCV, NWBV, or visual ratings: MTA, GCA), CSF biomarkers (Aβ, Tau), or both to the model. The models with volumetric MRI measures were adjusted for field strength. In all analyses, we intensively investigated main effects of patient characteristics and interaction effects between biomarkers and between biomarker and patient characteristics. Effects were retained in the model via a backward selection procedure, if *p* value ≤ .10. The prognostic accuracy of the model was measured by Harrell’s *C*-statistic.

We estimated cumulative progression probabilities with 95% confidence intervals using the survci command in STATA [35]. We report 5-, 3-, and 1-year cumulative progression probabilities with corresponding confidence intervals. Since the clinical follow-up visit times showed some variation, the cutoff for 1-year follow-up was set at 1.5 years, for 3-year follow-up at 3.5 years, and for 5-year follow-up at 5.5 years. As an example, we provide risk estimates for individuals with an age of 55 and 70; females and males and MMSE scores of 30 or 27. To contrast individuals with normal and abnormal MRI and CSF results, we entered 10th and 90th percentile MRI and CSF values in the Cox model. Note that when using the models, any value can be entered for a variable. Based on the constructed models, 5-year progression probabilities were calculated for every patient in the cohort. Subsequently, we labeled each individual as having low risk (≤ 35%), intermediate risk (35–65%), or high risk (> 65%).

In an additional set of analyses, we repeated all analyses to construct models predicting progression to MCI or AD dementia as clinical end-point. In this set of analyses, individuals progressing to non-AD were censored at time of diagnosis of non-AD dementia.

### Validation

We internally validated the models by five-fold cross-validation, in which we again applied a backward selection procedure.

Next, we performed external validation of our models on a sample comprising individuals with SCD from Alzheimer’s Disease Neuroimaging Initiative (ADNI, *n* = 92), Dementia Competence Network (DCN, *n* = 86), and Barcelona Memory Clinic (*n* = 41). Like the ADC cohort, DCN and Barcelona included individuals that went to the memory clinic to seek medical help and were labeled with SCD when cognitive testing could not confirm their cognitive complaints, and criteria for MCI, dementia, or other neurological or psychological diseases were not met. ADNI on the other hand is a population-based study. Subjects were labeled with SCD when a significant subjective memory concern was reported by the subject, informant, or clinician.

CSF was measured with Innotest in the DCN and Barcelona cohort and with Elecsys in ADNI. Therefore, biomarker values were standardized for the analysis to remove measurement levels. Patient characteristics from the cohorts can be found in Additional file 1: Table S2. Differences between the cohorts included a higher age in the ADNI cohort, higher progression rates in the DCN, longer follow-up for ADC and Barcelona individuals; and ADNI and Barcelona individuals were more often female (Additional file 1: Table S2). Established models were fitted to the validation data, and Harrell’s *C*-statistics were calculated.

## Results

During a mean follow-up of 3 ± 2 years, 70 (15%) individuals showed clinical progression to MCI (*n* = 49), AD dementia (*n* = 10), or non-AD dementia (*n* = 11). Mean age was 62 ± 9 years, 211 (44%) of the individuals were female, and the mean MMSE score was 28 ± 1.6 (Table 1).

**Table 1:** Baseline characteristics

	SCD individuals*n* = 481
No. (%) with clinical progression	70 (15%)
Progression to MCI	49 (10%)
Progression to AD dementia	10 (2%)
Progression to non-AD dementia	11 (2%)
Age	62 ± 9
Gender, no. (%) females	211 (44%)
MMSE	28 ± 1.6
Follow-up duration	3 ± 2
Medial temporal lobe atrophy	0.4 ± 0.5
Global cortical atrophy	0.4 ± 0.6
Hippocampal volume (cm3)	7.2 ± 1
Normalized whole brain volume (cm^3^)	1453 ± 100
Amyloid β1–42	879 ± 260
Total Tau	298 ± 196
p-tau	49 ± 22

Data are mean ± standard deviation, unless otherwise specified. *MMSE* mini-mental state examination, *MRI* magnetic resonance imaging, *CSF* cerebrospinal fluid

Table 2 shows the variables and corresponding coefficients included in the models (demographics only, CSF model, MRI volumetric model, and MRI visual model).

**Table 2:** Regression coefficient of the final model

	MCI/dementia				MCI/AD-dementia			
	Coëfficient	Standard error	*p* value	Harrell’s *C*	Coëfficient	Standard Error	*p* value	Harrell’s *C*
Demographic (*n* = 481)								
Age	0.0854	0.0147	< .001	0.70	0.0904	0.0162	< .001	0.70
MMSE	− 0.2497	0.0639	< .001		− 0.2236	0.0726	< .01	
CSF (*n* = 344)								
Aβ	− 1.0462	0.3668	< .01	0.82	− 1.3941	0.4031	< .01	0.84
Tau	1.2785	0.3384	< .001		1.3829	0.4003	< .01	
Age	0.0704	0.0251	< .01		0.0644	0.0296	< .05	
Gender	− 0.6360	0.3345	< .10		− 0.6375	0.3814	< .10	
MMSE	− 0.25503	0.0815	< .01		− 0.1846	0.0981	< .10	
Tau* age	− 0.1393	0.0467	< .01		− 0.1311	0.0534	< .05	

CSF biomarkers (Aβ and Tau) are log-transformed and centered. *MMSE* mini-mental state examination, Tau* age interaction between age and Tau. Interaction term was centered and standardized to allow inclusion in the model

The demographics only model included age and MMSE (sex not included, *p* value > .10). Harrell’s *C*-statistic was 0.70 (Table 2). Younger individuals (as an example, we set age at 55) with MMSE-scores of 30 had a low risk of progression: after 5 years 6% [3–11], after 3 years 4% [2–8], and after 1 year 2%[0–2]. On the other end of the spectrum, older individuals (70) with lower MMSE-scores (27) had higher progression probabilities; risk of progression after 5 years was 38% [29–49], after 3 years 28% [21–37], and after 1 year 11% [7–16] (Table 3).

**Table 3:** Progression probabilities after 1 year, 3 years, and 5 years

				Demographics only	CSF			
	Age	Sex	MMSE		Normal	AB abnormal	Tau abnormal	Both abnormal
1 year	55	m	30	2% [0–2]	0% [0–1]	0% [0–2]	12% [3–38]	26% [7–71]
			27	3% [2–5]	0% [0–2]	0% [1–5]	26% [8–65]	51% [18–92]
		f	30	2% [0–2]	0% [0–1]	0% [0–1]	6% [2–23]	15% [4–48]
			27	3% [2–5]	0% [0–1]	0% [1–3]	15% [4–44]	32% [9–77]
	70	m	30	5% [3–9]	5% [2–15]	13% [5–34]	4% [1–11]	8% [3–20]
			27	11% [7–16]	12% [5–30]	29% [11–64]	9% [3–21]	19% [9–36]
		f	30	5% [3–9]	3% [1–9]	7% [2–22]	3% [1–6]	5% [2–12]
			27	11% [7–16]	7% [2–22]	16% [5–46]	5% [2–12]	10% [4–24]
3 years	55	m	30	4% [2–8]	0% [0–2]	1% [1–6]	32% [10–78]	60% [20–98]
			27	9% [6–14]	1% [0–5]	2% [0–12]	60% [23–95]	89% [44–99]
		f	30	4% [2–8]	0% [0–1]	1% [0–3]	19% [5–55]	39% [11–87]
			27	9% [6–14]	0% [0–3]	1% [0–7]	39% [12–84]	69% [26–99]
	70	m	30	15% [10–21]	16% [6–39]	35% [14–72]	13% [4–27]	23% [11–46]
			27	28% [21–37]	33% [14–71]	64% [30–96]	24% [10–49]	48% [28–71]
		f	30	15% [10–21]	9% [3–25]	21% [7–53]	7% [3–16]	16% [6–29]
			27	28% [21–37]	21% [7–53]	43% [15–85]	14% [5–32]	29% [14–50]
5 years	55	m	30	6% [3–11]	1% [0–3]	1% [0–8]	43% [13–89]	73% [27–98]
			27	12% [8–20]	1% [1–7]	4% [1–17]	73% [30–99]	96% [56–100]
		f	30	6% [3–11]	0% [0–2]	1% [0–4]	25% [7–69]	51% [16–94]
			27	12% [8–20]	1% [0–4]	2% [0–10]	50% [17–93]	81% [35–98]
	70	m	30	20% [14–29]	22% [9–51]	47% [19–84]	17% [6–36]	32% [16–58]
			27	38% [29–49]	44% [19–83]	78% [39–98]	32% [24–62]	60% [38–83]
		f	30	20% [14–29]	13% [4–34]	28% [10–66]	10% [5–22]	21% [8–38]
			27	38% [29–49]	28% [10–66]	55% [20–94]	20% [10–39]	39% [20–66]

Biomarker values were selected as 90th percentile (normal, −) and 10th percentile (abnormal, +); for Tau, 10th percentile was selected as normal (−) and 90th percentile as abnormal (+). Note that this table is an example, as the model can provide individualized risk estimates for any given value. Data are % [95% CI]. For CSF, −/− AB and Tau negative: +/− AB positive and Tau negative; −/+ AB negative and Tau positive; +/+ AB and Tau positive

**Fig. 1:**
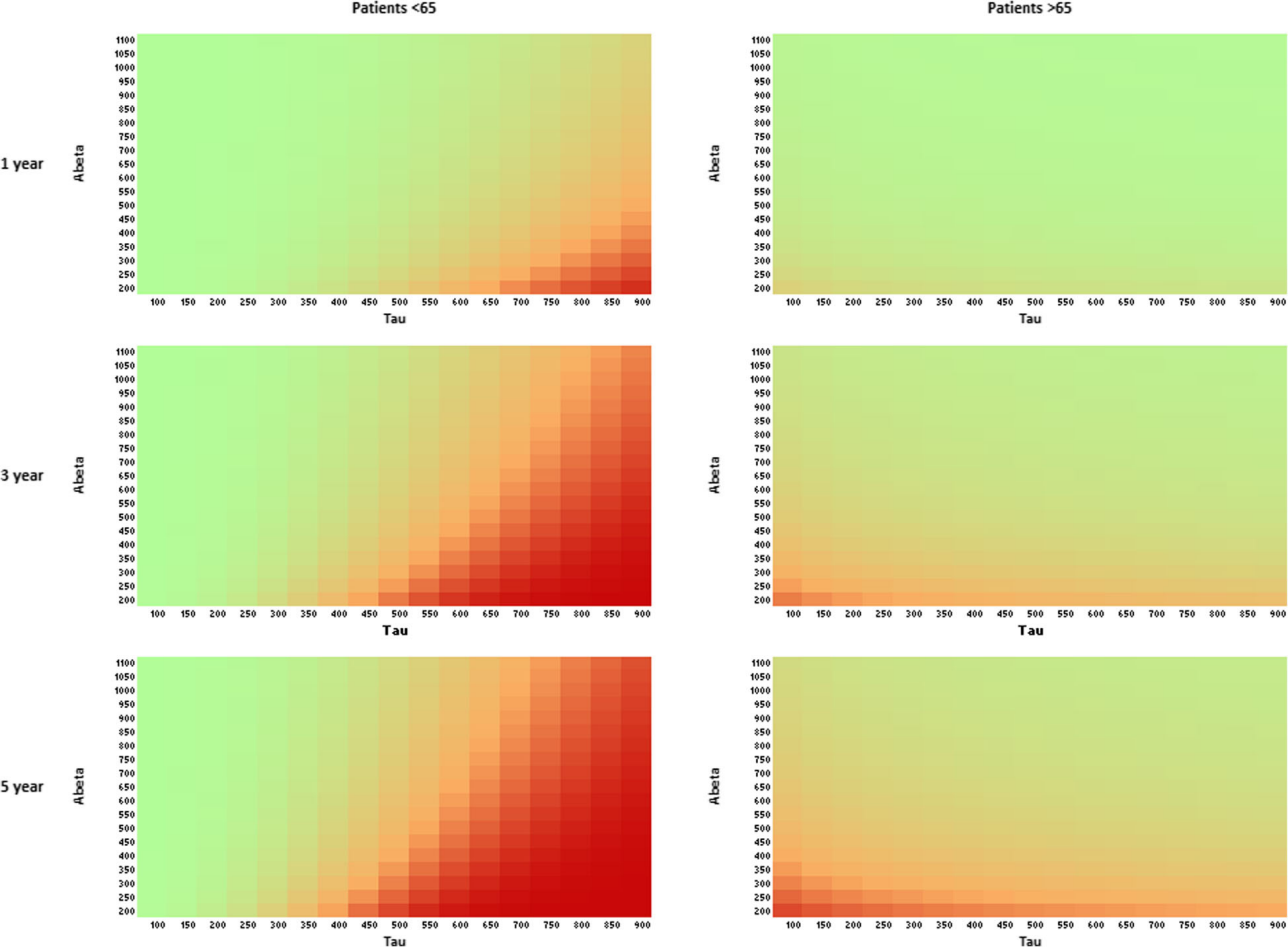
Probability isographs for 1-, 3-, and 5-year progression to MCI or dementia. Legend: Probability of progression within 1 (upper panel) year, 3 (middle panel) years, and 5 (lower panel) years based on Aβ (pg/mL, *y*-axis) and Tau (pg/mL, *x*-axis), stratified for individuals younger (left) and older than 65 (right)

When we evaluated MRI markers, neither volumetric nor visual measures added predictive value over the demographic model including age and MMSE (*p* value > .10). In the CSF model, female gender, higher age, lower MMSE score, lower Aβ, and higher Tau values were predictive of progression. Moreover, an interaction between Tau and age is retained in the model. Tau was more predictive than Aβ, especially in younger individuals (Tau* age *p* value < .01, Table 3 and Fig. 1). Harrell’s *C*-statistic was 0.82 (Table 2, similar when p-tau was included instead of tau (Additional file 1: Table S3). To contrast individuals with normal and abnormal CSF results, we derived probabilities for individuals with 10th and 90th percentile CSF values from the model.

Abnormal Aβ and Tau resulted in high 5-, 3-, and 1-year progression risks; 96% [56–100], 89% [44–99], and 51% [18–92]. By contrast, normal CSF biomarkers strongly decreased progression probabilities to 1% [0–3] in 5 year, 0% [0–2] in 3 years, and 0% [0–1] in 1 year, indicating the negative predictive value of these biomarkers. Please note that we report examples, as the model provides risks for any given value.

**Fig. 2:**
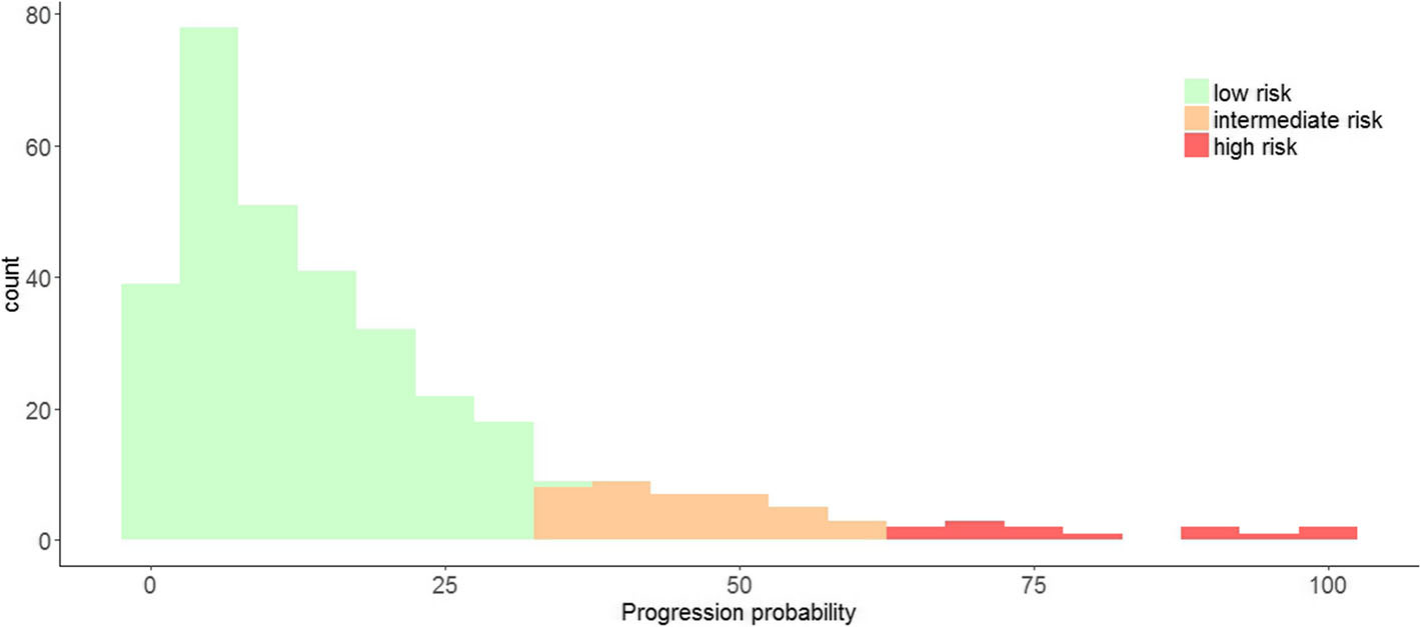
Distribution of 5-year progression probabilities. Legend: Distribution of 5-year progression probabilities based on the CSF model. Green: low risk 0–35%; orange: intermediate 35–65%; red: high risk 65–100%

Figure 2 shows the distribution of 5-year progression probabilities based on the model including CSF biomarkers. The majority of individuals, 84% (*n* = 290) were labeled as having low risk of progression, 12% (*n* = 41) had intermediate risk of progression, and 4% (*n* = 13) had high risk of progression. Of note, 58% of the individuals that were classified as “intermediate” based on the demographic model could be reclassified as having low (49%) or high (9%) risk according to the CSF model (Additional file 1: Table S4).

In an additional set of analyses, we repeated the analysis restricting the outcome to progression to MCI or AD dementia. The prognostic accuracy of the CSF model increased in line with specificity for AD of the biomarkers under evaluation. Harrell’s *C*-statistic remained 0.70 for the demographic model and increased to 0.84 for the CSF model.

Internal validation by five-fold cross-validation confirmed prognostic performance in both models (Additional file 1: Table S5); cross-validation of the demographic model resulted in Harrell’s *C*-statistics ranging from 0.63–0.77. The model with CSF biomarkers showed cross-validated Harrell’s *C* ranging from 0.75–0.90.

External validation showed moderate performance of the models (demographic model, Harrell’s *C* = 0.62; CSF model, *C* = 0.68).

## Discussion

In this study, we constructed biomarker prediction models that provide individual risk estimates of clinical progression in order to optimize the interpretation of biomarkers for cognitively normal SCD individuals. CSF biomarkers considerably improved prognostic performance over the use of age and MMSE only. This was mostly driven by their strong negative predictive value.

Alzheimer pathology as reflected in biomarker changes presumably starts more than 20 years before the onset of dementia [3, 8]. Clinicians are reluctant to disclose biomarker results to cognitively normal individuals presenting at a memory clinic, as former findings that were based on group level cannot directly be translated to the individual. Moreover, there is always a degree of uncertainty associated with the interpretation of biomarkers. With our models, we provide a first step towards a framework for a personalized approach, allowing the use of biomarker results for cognitively normal individuals presenting at memory clinics. This can be useful, as individuals and caregivers become increasingly assertive in their need for information on their risk of dementia. Moreover, interest in individualized risk profiling and both primary and secondary prevention strategies is increasing rapidly. Although truly longitudinal data are lacking, our sample allowed to infer predictions of progression over periods of 3 and even 5 years, which has great relevance for individuals and their family members. Probabilities of progression within 1 year in SCD individuals remain low, and this is in line with the notion that outcome at 1-year follow-up is not a reasonable time frame for SCD, as these individuals initially perform cognitively normal.

Former studies have shown the clinical relevance of CSF biomarkers in pre-dementia individuals on a group level [15, 16]. In the current study, we found that Tau was a stronger predictor than Aβ. Particularly, Tau was more predictive for progression in younger individuals. Abnormal Tau values in older individuals were less predictive, probably due to normal aging processes or multiple pathologies in older individuals [36]. Moreover, gender was included as a predictor in the CSF model, meaning that CSF measures should also be interpreted in the context of a patient’s gender. This fits with findings from a recently published review that showed the importance of sex differences for patient stratification and personalized treatment [37]. With these CSF biomarkers and patient characteristics, 88% of the individuals could be classified as having a high (> 65%) or low (≤ 35%) risk of clinical progression within 5 years.

Former studies on MRI biomarkers have reported that cognitively normal individuals with SCD had lower hippocampal volumes compared to healthy controls [11]. Moreover, hippocampal atrophy and lower brain volumes predicted of progression to MCI and/or dementia [10, 38]. However, these previously reported significant results for MRI were mostly based on small absolute differences between groups of individuals, precluding their usefulness in individualized risk predictions. In the current study, MRI markers did not improve personalized risk estimates over the use of age and MMSE only. The effects of MRI biomarkers lost significance, as soon as age was included in the model, suggesting that the observed atrophy in this population is largely attributable to aging and/or did not capture additional predictive value over subtle cognitive impairment.

In a former study in MCI, we found that MRI biomarkers in combination with patient characteristics and also CSF biomarkers improve individualized prediction of progression to dementia [4]. The finding that atrophy on MRI has less predictive value in cognitively normal individuals than in MCI patients is consistent with the hypothetical biomarker model which suggests that CSF biomarker changes precede MRI-based estimates of neurodegeneration [39].

Among the limitations, we found that the models showed somewhat less prognostic performance in external cohorts. This may be attributable to the fact that the outcome in the current study is clinical progression to MCI or dementia. While dementia is a relatively definitive end-point, MCI patients may still remain stable or convert to normal states of cognition and variability in this diagnosis between centers may be larger than in case of dementia [3, 24, 40]. In addition, external validation is highly dependent on the case-mix of a sample. In the field of SCD, one of the most important unresolved challenges is the variability of defining SCD across studies [41–43]. In the ADC, DCN, and Barcelona cohort, all individuals went to the memory clinic seeking help and their complaints might be more severe than in the population-based ADNI cohort. In ADNI, individuals were labeled with SCD when a significant subjective memory concern was reported by the subject, informant, or clinician. Moreover, the standardized diagnostic work-up differed between the centers. For example, ADNI measured CSF with Elecsys instead of Innotest and brain volume with Freesurfer software instead of FSL FIRST. However, we limited these differences as much as possible by standardizing the biomarker values to remove measurement levels.

Another limitation is that we used different scanners and field strengths. This however resembles real-life clinical practice, and we included field strength as an additional determinant in the models. Field strength, however, did not improve predictive ability of MRI models. In addition, we used MMSE as a measure of global cognition in our models, which has been described as an insensitive instrument in preclinical stages. Other measures for cognition (for example, a composite score of specific items of the MMSE and Clinical Dementia Rating scale; ADCOMS [44], or instrumental activities of daily living (IADL [45])) with higher sensitivity could improve the models. Such an approach might be subject for future studies. Lastly, the follow-up duration varied between individuals and the mean follow-up period of 3 ± 2 years was rather short in comparison with the assumed duration of the stage of preclinical AD. Nonetheless, we had enough power to estimate risks over a period of 5 years, which is a considerable duration of follow up.

A major strength of this study is the simplicity of the models. Often, the goal of constructing prediction models is to derive the most optimal combination of many variables. However, such models often require multiple pieces of data that are not easily available, consequently limiting their clinical footprint [46]. In the current paper, we took a different approach as we aimed to optimize the interpretation of MRI measures and CSF biomarkers in individuals with SCD: given that a clinician has decided to obtain MRI and/or CSF biomarkers in an individual with SCD with a given age, sex, and MMSE, this clinician wants to make optimal use of the results of MRI and CSF. By doing so, our models helps to interpret biomarkers in the individual patient (hence personalized) and shows proof of principle that personalized predictions could be feasible in very early stages of AD. Another strength is that we used a large sample of SCD individuals. All individuals had an extensive screening at baseline to rule out MCI or other neurological causes of memory complaints and careful follow-up, with diagnosis re-evaluated in a multidisciplinary setting, which has contributed to the robustness of the data. The vast majority of individuals came to the memory clinic with worries about their cognitive functioning, rendering our models highly relevant for this population. In fact, this population is comparable to what in the new Alzheimer research framework is described as clinical stage 2 [8]. Our results confirm clinical validity of such stage 2, as the presence of Alzheimer biomarkers strongly increases the risk of future progression to MCI or dementia. Another strong aspect of this study is that we accompany predictions with confidence intervals, which gives a good indication on precision of the prediction.

In an earlier study on communication of diagnostic test results, individuals and caregivers who recently visited a memory clinic indicated that they wanted more information on their prognosis and what test results meant for their personal lives (“what do these results mean for my future”) [47]. Nonetheless, clinicians tend to be reluctant to disclose biomarker results to cognitively normal individuals. The major concern is that disclosure could increase anxiety [48]. An argument against the disclosure of risk is the lack of treatment options. But this raises the question whether it is ethical to withhold individuals from information that is actually available. Moreover, our models show that particularly the negative predictive value of the models is good, suggesting that biomarker results can be especially valuable to reassure patients.

## Conclusions

In conclusion, we constructed prognostic models that allow interpretation of biomarker data in cognitively normal individuals in a memory clinic at the individual level. In light of future disease-modifying drugs, risk prediction on an individual level becomes increasingly important [49]. By integrating biomarker results and demographic characteristics in AD risk modeling, the current study takes the first steps in a personalized approach for cognitively normal individuals [48, 50]. This is especially valuable for the reassurance of individuals with normal biomarkers, since clinical progression over a period of 5 years is very unlikely for them.

## Additional file

Additional file 1: **Figure S1.** Flowchart patient selection. **Figure S2.** Kaplan-Meier based on CSF risk group. **Table S1.** Demographic characteristics and baseline survival for complete cases and incomplete cases. **Table S2.** Baseline patient characteristics validation cohort. **Table S3.** Regression coefficient of CSF model including p-tau. **Table S4.** Reclassification table based on the demographic and CSF model. **Table S5.** Model development by internal cross validation. **Table S6.** Model development in the validation cohort. (DOCX 84 kb)
