# Supplementary material for: Personalized risk for clinical progression in cognitively normal subjects—the ABIDE project
Source: Alzheimers Res Ther. 2019 Apr 16;11:33. doi: 10.1186/s13195-019-0487-y (PMC6466790; doi:10.1186/s13195-019-0487-y)
Supplement: Supplementary file 1 — Figure S1. Flowchart patient selection. Figure S2. Kaplan-Meier based on CSF risk group. Table S1. Demographic characteristics and baseline survival for complete cases and incomplete cases. Table S2. Baseline patient characteristics validation cohort. Table S3. Regression coefficient of CSF model including p-tau. Table S4. Reclassification table based on the demographic and CSF model. Table S5. Model development by internal cross validation. Table S6. Model development in the validation cohort. (DOCX 84 kb) [file 13195_2019_487_MOESM1_ESM.docx]

Supplementary Material

**Personalized risk for clinical progression in cognitively normal subjects – the ABIDE project**

I.S. van Maurik^1,2*^, R.E.R. Slot, MD^1^, S.C.J. Verfaillie^1^, M.D. Zwan, PhD^1^, F.H. Bouwman, MD, PhD^1^, N.D. Prins, MD, PhD^1,3^, C.E. Teunissen, PhD^4^, P. Scheltens, MD, PhD ^1^, F. Barkhof, MD, PhD^5,6^, M.P. Wattjes, MD, PhD^5^, J.L. Molinuevo, MD, PhD^7^ , L. Rami, MD, PhD^7^, S. Wolfsgruber, PhD^8^, O. Peters, MD, PhD^9^, F. Jessen, MD, PhD^10^, J. Berkhof, PhD^2^, W.M. van der Flier, PhD^1,2^, for the Alzheimer’s Disease Neuroimaging Initiativeᶧ

Supplementary eFigure 1. Flowchart patient selection

Supplementary eFigure 2. Kaplan-Meier based on CSF risk group

Supplementary eTable 1. Demographic characteristics and baseline survival for complete cases and incomplete cases

Supplementary eTable 2. Baseline Patient characteristics validation cohort

Supplementary eTable 3. Regression coefficient of CSF model including p-tau

Supplementary eTable 4. Reclassification table based on the demographic and CSF model

Supplementary eTable 5. Model development by internal cross validation

Supplementary eTable 6. Model development in the validation cohort

**Supplementary eFigure 1. Flowchart patient selection**
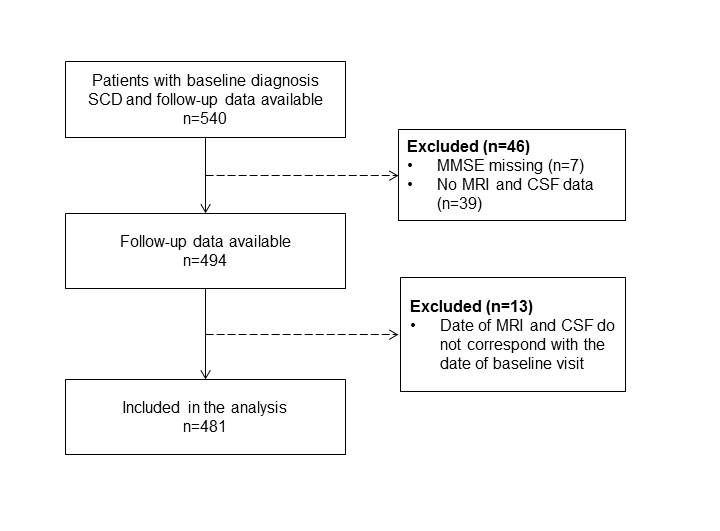


**Supplementary eFigure 2. Kaplan-Meier based on CSF risk group**


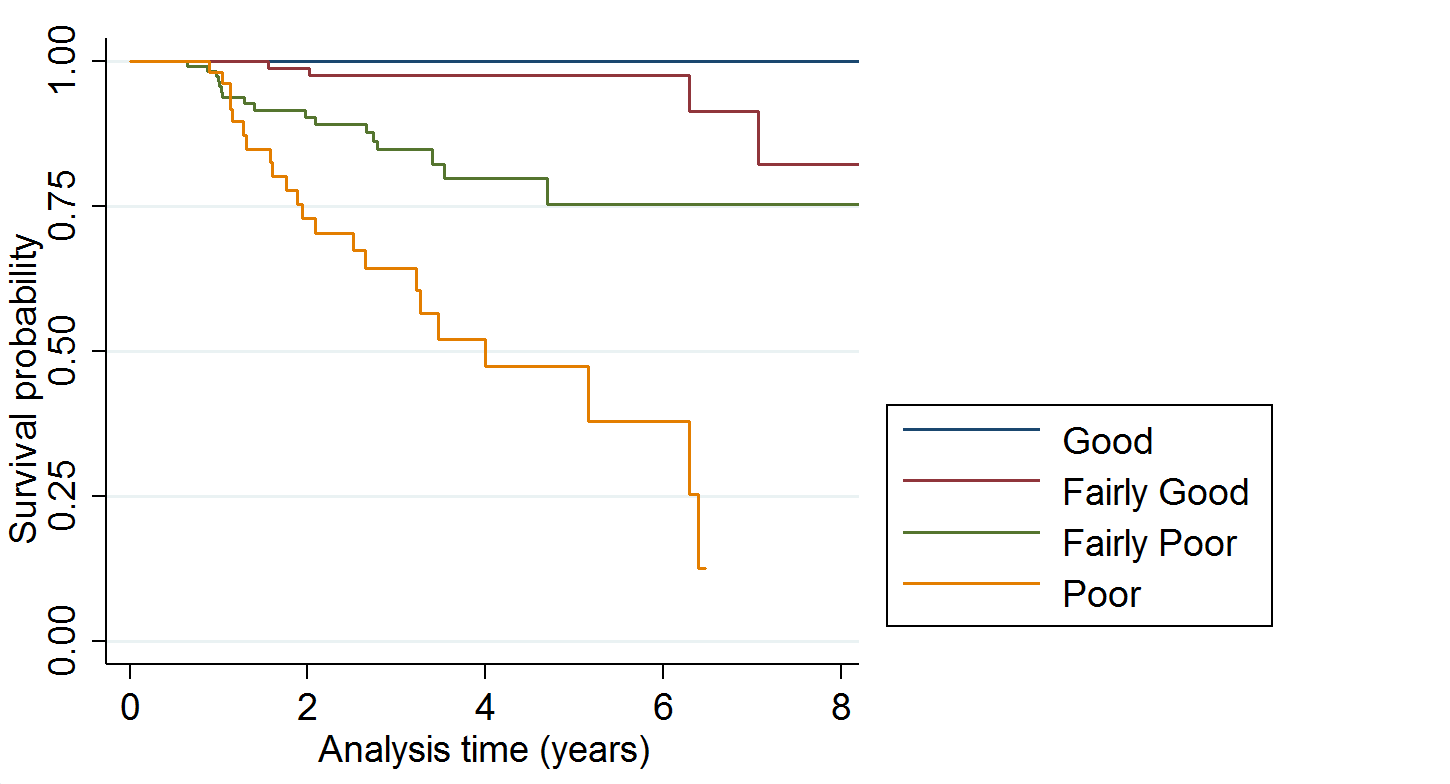


Risk groups were based on the 16^th^, 50^th^ and 84^th^ percentile of the prognostic index based on the CSF model, resulting in a Good, Fairly Good, Fairly Poor and Poor risk group.

**Supplementary eTable 1. Demographic characteristics and baseline survival for complete cases and incomplete cases**

|  | **Complete data** | **Only MRI data** | **Only CSF data** | **p-value** |
| --- | --- | --- | --- | --- |
| Age | 61±9 | 64±10 | 61±10 | 0.005 |
| Gender, No. females | 120 (41) | 72 (52) | 19 (40) | NS |
| MMSE | 28±2 | 28±2 | 28±2 | NS |
| No. with clinical progression (%) | 41 (14) | 24 (17) | 5 (11) | NS |
| Baseline Survival function | 0.90 | 0.91 | 0.92 | NS |

Data are mean±SD, unless otherwise specified. MMSE: mini-mental state examination

**Supplementary eTable 2. Baseline Patient characteristics validation cohort**

|  | **ADC**  **n=481** | **ADNI n=92** | **DCN**  **n=86** | **Barcelona**  **n=41** |
| --- | --- | --- | --- | --- |
| No. with clinical progression (%)ᶧ | 70 (15%) | 9 (10%) | 33 (38%) | 5 (12%) |
| Ageᶧ | 62±9 | 72±6 | 65±8 | 66±7 |
| Gender, No. femalesᶧ | 211(44%) | 55 (60%) | 25(29%) | 32 (78%) |
| MMSE | 28±2 | 29±1 | 28±2 | 28±2 |
| Follow-up durationᶧ | 3±2 | 2±1 | 2±1 | 4±2 |
| Amyloid β1-42* | 879±260 | 1249±443 | 778±314 | 754±220 |
| Total Tau* | 298±196 | 238±93 | 347±225 | 372±322 |

Data are mean±SD, unless otherwise specified. MMSE: mini-mental state examination, MRI=magnetic resonance imaging, CSF=cerebrospinal fluid. *****measured with Elecsys for ADNI, measured with Innotest for DCN and EUROSCD. Differences between cohorts were mearured with chi-square and ANOVA where applicable. Due to difference in measurement levels, CSF values were logtransformed and centered. ᶧ indicates p<0.001.

**Supplementary eTable 3. Regression coefficient of CSF model including p-tau**

|  | **MCI/Dementia** | | |  |  | **MCI/AD-dementia** | | |  |
| --- | --- | --- | --- | --- | --- | --- | --- | --- | --- |
|  | **Coëfficient** | **Standard Error** | **P-value** | **Harrell's C** |  | **Coëfficient** | **Standard Error** | **P-value** | **Harrell's C** |
| **CSF**  **(n=344)** |  |  |  |  | **CSF**  **(n=344)** |  |  |  |  |
| Aβ | -1.1592 | 0.3746 | <.01 | 0.80 | Aβ | -1.5333 | 0.4296 | <.001 | 0.83 |
| p-tau | 1.0945 | 0.4073 | <.01 |  | p-tau | 1.2253 | 0.4785 | <.01 |  |
| Age | 0.0650 | 0.0228 | <.01 |  | Age | 0.0603 | 0.0269 | <.05 |  |
| Gender | -0.6027 | 0.3346 | <.10 |  | MMSE | -0.2240 | 0.0966 | <.05 |  |
| MMSE | -0.2762 | 0.0772 | <.001 |  | p-tau*age | -0.1096 | 0.0580 | <.10 |  |
| p-tau*Age | -0.1251 | 0.0504 | <.05 |  |  |  |  |  |  |

CSF biomarkers (Aβ and p-tau) are log-transformed and centered. MMSE: mini-mental state examination, p-tau*Age: interaction between age and p-tau.

**Supplementary eTable 4. Reclassification table based on the demographic and CSF model**

|  |  | Demographic model | | |  |
| --- | --- | --- | --- | --- | --- |
|  |  | low | intermediate | high |  |
| CSF model | low | 278 (93%) | 22 (49%) | 0 (0) | *300* |
|  | intermediate | 17 (6%) | 19 (42%) | 0 (0) | *36* |
|  | high | 3 (1%) | 4 (9%) | 1 (100%) | *8* |
|  |  | *298* | *45* | *1* |  |

Reclassification of individuals based on the demographic model and CSF model. Low risk: 0-35%, intermediate risk: 35-65%, high risk: 65-100%.

**Supplementary eTable 5. Model development by internal cross validation**

|  |  | **Demographic only** | | **CSF** | |
| --- | --- | --- | --- | --- | --- |
| **Outcome** | **Set** | **c** | **best fit model** | **c** | **best fit model** |
| MCI/Dementia | 1 | 0.75 | Age, MMSE | 0.82 | Abeta, Tau, Age, MMSE, Tau*Age |
|  | 2 | 0.70 | Age, MMSE | 0.75 | Abeta, Tau, Age, MMSE, Tau*Age, Tau*MMSE |
|  | 3 | 0.77 | Age, MMSE | 0.90 | Abeta, Tau, Age, Sex, MMSE Tau*Age |
|  | 4 | 0.69 | Age, MMSE | 0.77 | Abeta, Tau, Age, MMSE, Tau*Age, Tau*MMSE |
|  | 5 | 0.63 | Age, MMSE | 0.77 | Abeta, Tau, Age, Sex, MMSE, Tau*Age, Tau*MMSE |

Cross validation was performed with a fivefold cross validated Harrell’s concordance index. The dataset was randomly split up in five groups, models were constructed via backward selection on the training set (four out of five subsets) and validated on the test set that was left out. The process was repeated five times.

**Supplementary eTable 6. Model development in validation cohort**

|  | **Coefficient** | **Standard Error** | **P-value** | **Harrell's C in validation cohort** | **Harrell's C in ADC** |
| --- | --- | --- | --- | --- | --- |
| **Demographic** |  |  |  |  |  |
| Age | 0.0469 | 0.0140 | 0.001 | 0.62 | 0.70 |
| MMSE | -0.1264 | 0.0590 | <0.05 |  |  |
|  |  |  |  |  |  |
| **MRI model** |  |  |  |  |  |
| HCV (cm3) | -0.7546 | 0.2284 | 0.001 | 0.74 | 0.55 |
| NWBV (cm3) | 0.0077 | 0.0020 | <0.001 |  |  |
| Age | 0.0278 | 0.0379 | 0.463 |  |  |
| HCV*Age | -0.0422 | 0.0249 | 0.090 |  |  |
|  |  |  |  |  |  |
| **CSF model** |  |  |  |  |  |
| Abeta | -0.7744 | 0.3523 | <0.05 | 0.68 | 0.75 |
| Tau | 0.4970 | 0.2345 | <0.05 |  |  |
| Gender | -0.9761 | 0.3182 | <0.01 |  |  |
| MMSE | -0.1889 | 0.0831 | <0.05 |  |  |
|  |  |  |  |  |  |
| **Combined model** |  |  |  |  |  |
| Abeta | -0.5333 | 0.5514 | 0.333 | 0.76 | 0.53 |
| NWBV | 0.0073 | 0.0020 | <0.001 |  |  |
| HCV | -0.8122 | 0.2463 | 0.001 |  |  |
| Age | 0.0139 | 0.0403 | 0.731 |  |  |
| MMSE | -0.0086 | 0.1409 | 0.952 |  |  |
| Abeta*MMSE | 0.4924 | 0.2799 | 0.078 |  |  |
| HCV*Age | -0.0444 | 0.0264 | 0.093 |  |  |

Models were developed in the validation cohort and validated in ADC. CSF biomarkers (Abeta and Tau) are log-transformed and centered. MMSE: mini-mental state examination, Abeta*MMSE: interaction between Abeta and MMSE.
